# Domestic Violence during Pregnancy and Women’s Health-Related Quality of Life

**DOI:** 10.5539/gjhs.v8n2p27

**Published:** 2015-06-01

**Authors:** Maryam Gharacheh, Shahdokht Azadi, Nooredin Mohammadi, Simin Montazeri, Zohre Khalajinia

**Affiliations:** 1 Department of Reproductive Health, Faculty of Nursing and Midwifery, Tehran University of Medical Sciences, Tehran, Iran; 2 Department of Psychology, Islamic Azad University, Gachsaran Branch, Gachsaran, Iran; 3 Centre for Nursing Research, Department of Critical Care Nursing, Faculty of Nursing and Midwifery, Iran University of Medical Sciences, Tehran, Iran

**Keywords:** domestic violence, pregnancy, abused women, quality of life

## Abstract

Domestic violence during pregnancy is a major health problem with significant psychological and physical impairments for pregnant women. To assess the relationship between domestic violence during pregnancy and women’s health-related quality of life (HRQoL), a cross-sectional study was conducted on 341 postnatal women who referred to urban health care centers in Gachsaran, Islamic Republic of Iran. Domestic violence was assessed using a questionnaire modified from the Abuse Assessment Screen (AAS), and Iranian version of Short Form-36 questionnaire was used to assess women’s HRQoL. The findings of the study showed 44.5% of women reported experiencing domestic violence during pregnancy. All the SF-36 subscales including both physical and mental health dimensions scored lower in the abused women compared to the non-abused women, and differences between the groups in the six subscales of SF-36 except ‘physical functioning’ and ‘bodily pain’ were statistically significant (P<.05). These results suggest that domestic violence during pregnancy is associated with poor HRQoL in abused women.

## 1. Introduction

Domestic violence is a global social and health problem, and has been recognized as one of the most common forms of gender-based violence (Mavrikiou, Apostolidou, & Parlalis, 2014; Stöckl et al., 2013). Worldwide, at least one in three women is beaten, forced to have sex, or otherwise abused in her lifetime (Akyüz, Yavan, Şahiner, & Kılıç, 2012). For many women, physical and sexual violence continues during pregnancy (Akyüz et al., 2012; Devries et al., 2010). A wide range of prevalence rates of domestic violence during pregnancy has been estimated from 3 to 30 percent (Van Parys, Verhamme, Temmerman, & Verstraelen, 2014). In Iran a high prevalence of domestic violence has also been reported. Iranian studies have revealed that 60.6 percent of women experience multiple types of domestic violence during pregnancy (Abadi, Ghazinour, Nojomi, & Richter, 2012; Jahanfar & Malekzadegan, 2007).

Pregnancy does represent a time of particular vulnerability to the potential negative impacts of domestic violence (Audi, Segall-Corrêa, Santiago, & Pérez-Escamilla, 2012). Physiological changes during pregnancy contribute to decline in the physical health status and reduce the capability of women to perform their daily roles (Haas et al., 2005). The distress which women experience during pregnancy can be overwhelmed by domestic violence and have more significant effects on the well-being of pregnant women (Groves, Kagee, Maman, Moodley, & Rouse, 2011).

Domestic violence is increasingly being identified as a significant risk factor for adverse health consequences for both mother and newborn (Devries et al., 2010). The experience of domestic violence during pregnancy is associated with numerous negative consequences including prenatal bleeding, fetal fractures, maternal infections, uterus, lung or spleen rupture, abortion, stillbirth and premature birth (Akyüz et al., 2012; Bailey, 2010). Adverse mental health outcomes and behavioral risks such as depression, anxiety, post-traumatic stress disorder, suicide attempts, delayed prenatal care, poor maternal nutrition and drug and alcohol abuse are consistently related to domestic violence during pregnancy (Van Parys et al., 2014). Hence, domestic violence appears to be an indicator of an increased risk of serious mortal danger for some pregnant women (Lau, Wong, & Chan, 2008). It can manifest as an indicator for poor health status, poor quality of life and high use of health services (Sørensen, Kruse, Gudex, Helweg-Larsen, & Brønnum-Hansen, 2012).

Some studies have shown that domestic violence has wide-ranging impacts on health-related quality of life (HRQoL) (Sørensen et al., 2012; Wittenberg, Joshi, Thomas, & McCloskey, 2007). In a study, Leung, Leung, Ng, and Ho (2005) found that the mean scores of HRQoL among the abused women were significantly lower compared with non-abused women (Leung, Leung, Ng, & Ho, 2005). HRQoL appears to be a good indicator to assess the life situation of women who experience domestic violence (Alsaker, Moen, & Kristoffersen, 2008).

HRQoL is a multi-dimensional concept, including domains related to physical, mental, emotional and social functioning, and describes the overall effect of a disease, illness or condition on the health of the individual (Bakas et al., 2012). According to the WHO definition, HRQoL is an “individual’s perception of their position in life in the context of the culture and value systems in which they live and in relation to their goals, expectations, standards and concerns” (Romero, Vivas-Consuelo, & Alvis-Guzman, 2013). HRQoL is a concern of policymakers, researchers, and health care professionals (Bakas et al., 2012), and it is increasingly applied to describe population health status and to evaluate outcome from health care interventions (Sørensen et al., 2012). Therefore, it is important to understand the HRQoL of women experiencing domestic violence for the baseline and serial assessment of these women (Leung et al., 2005) to improve health care and the cost-effectiveness of interventions (Sadler, Booth, Nielson, & Doebbeling, 2000).

Although some studies have reported a relationship between the experience of domestic violence and the significant impairment of HRQoL among women (Leung et al., 2005; Sørensen et al., 2012), the extent of this association among pregnant women is not clear. Thus, this study aimed to explore the relationship between domestic violence during pregnancy and women’s HRQoL.

## 2. Methods

This cross-sectional study was conducted from July 2012 to December 2012 on women who referred to health care centers for the postnatal care (during the eight weeks after delivery) in Gachsaran city, Islamic Republic of Iran. A convenience sample of 341 women was collected from urban health care centers based on the covered population. The inclusion criteria were: married women with Iranian nationality, and age between 20 and 39 years. Exclusion criteria were: having any complications with the mother or the baby, and having any history of medical or mental disorder. After removal of incomplete forms, 328 cases remained in the study.

Domestic violence during pregnancy was assessed using a questionnaire, culturally modified from Abuse Assessment Screen (AAS). Some questions were added to this form regarding different types of domestic violence. After determining its validity, its reliability was evaluated by coefficient Cronbach’s alpha, and the obtained correlation coefficient was 0.85. The abuse assessment questionnaire consisted of questions regarding socio-demographic data and three sections about experiences of physical (13 items), sexual (7 items) and emotional (15 items) violence. Physical violence was defined as the use of physical force against women that leads to physical or psychological harm including beating, kicking, stabbing, slapping, shooting, pushing, biting, pinching and/or choking. Sexual violence referred to any action and behavior that coerces women to do something of a sexual nature they don’t want to do. Emotional violence was defined as any act including confinement, isolation, verbal assault, intimidation, humiliation, or any other behavior which makes women feel diminished or embarrassed.

Iranian version of Short Form-36 (SF-36) Questionnaire was used to assess women’s HRQoL. The SF-36 is a reliable and valid instrument of functional status and is widely applied in health outcomes research (Sadler et al., 2000). This questionnaire in Iranian population was validated by Montazeri, Goshtasebi, Vahdaninia, and Gandek (2005). Its reliability was measured by the Cronbach’s alpha coefficients ranging from 0.77 to 0.90. The SF-36 comprises 36 items, divided into two domains of ‘physical health’ and ‘mental health’ with eight subscales including physical functioning, bodily pain, role limitations due to physical problems, role limitations due to emotional problems, social functioning, vitality, mental health and general health. Each scale ranged from 0 to 100 with lower scores, indicating poorer functioning.

After obtaining informed consent and describing the aim of the study, the participants were asked to complete the questionnaires. The questionnaires were self-administered in a private room without their husbands present. If a woman answered positively to any item of domestic violence, she was considered to be abused and those who answered no to all items of violence comprised the non-abused group. In the present study, the experience of domestic violence was only considered during pregnancy.

This study was approved by the Research Ethics Committee affiliated with Islamic Azad University, Gachsaran Branch. Participation in the study was voluntary, and confidentiality was maintained by anonymous completion of the questionnaires.

Statistical analysis was conducted using SPSS, version 16. The mean scores of HRQoL dimensions were calculated in both abused and non-abused groups. The mean differences between groups were analyzed using the ANOVA, and t-test. In addition, categorical variables were analyzed by chi-square test, and linear regression analysis was used to test the correlation between domestic violence and HRQoL scores. A p-value of less than .05 was considered statistically significant.

## 3. Results

A total of 146 women (44.5%) reported experiencing domestic violence during pregnancy. Of the abused women 88.4% experienced emotional violence, 34.9% sexual violence, and 26% physical violence. 67.8% of abused women experienced only one type of violence, 16.4% two different types of violence, and 15.8% three different types of violence. The mean age of abused women was 26.25±4.12 years, and the non-abused women had a mean age of 27.14±4.29 years. Most of the women (79%) had a high school level of education and most (94%) were unemployed. Socio-demographic variables were not significantly different between the abused and the non-abused women (P>.05) (Table 1).

**Table 1:** Socio-demographic characteristics in the abused and the non-abused women

Demographic characteristics	Non-abused N=182	Abused N=146	Significant level
	*M(SD)*	*M(SD)*	
Woman’s age (year)	27.14(4.29)	26.25(4.12)	P=.512
Husband’s age (year)	31.03(5.10)	30.86(4.76)	P=.487
Number of children	1.86(0.84)	1.83(0.93)	P=.073
Duration of marriage (year)	5.17(2.40)	4.78(2.33)	P=.531

The comparison of mean scores of HRQoL subscales in the abused and the non-abused women is presented in Table 2. Results showed that all the SF-36 subscales including both physical and mental health dimensions scored lower in the abused women compared to the non-abused women and differences between the groups in the six subscales of SF-36 except ‘physical functioning’ and ‘bodily pain’ were statistically significant (P<.05). The participants reported higher scores in the physical functioning subscale. The lowest score was demonstrated in the subscale of vitality (Table 2).

**Table 2:** Mean scores of HRQoL in the abused and the non-abused women

Mean scores of HRQoL	Non-abused N=182	Abused N=146	Significant level
	*M(SD)*	*Mean(SD)*	
Physical functioning	70.96(12.71)	69.10(13.70)	P=.210
Physical role	59.45(17.69)	54.93(21.39)	P=.041
Bodily pain	60.26(19.88)	57.59(17.82)	P=.202
General health	61.65(21.88)	54.43(21.02)	P=.003
Total physical health	62.83(15.91)	58.13(16.09)	P=.009
Vitality	57.47(18.26)	52.39(17.48)	P=.011
Social functioning	64.04(23.08)	56.88(22.35)	P=.005
Emotional role	59.60(27.69)	52.90(29.68)	P=.037
Mental wellbeing	65.61(19.38)	61.29(17.42)	P=.035
Total mental health	61.83(20.31)	55.71(19.99)	P=.007

Results of mean HRQoL scores of different types of domestic violence compared to women with no domestic violence are presented in Table 3. A linear trend of significantly declining SF-36 scores was observed in the subscale of ‘general health’, ‘vitality’, ‘social functioning’, ‘emotional role’, ‘total physical health’, and ‘total mental health’ (p<0.05) as the number of types of violence rose, so that women who had experienced three different types of domestic violence had the greatest HRQoL impairment. (Table 3)

**Table 3:** Mean scores of HRQoL of number of different types of domestic violence compared to women with no domestic violence

Types of violence	No violence experienced N=182	One type of violence experienced N=99	Two types of violence experienced N=24	Three types of violence experienced N=23	Significant level
HRQoL subscales	*M (SD)*	*M (SD)*	*M (SD)*	*M (SD)*	
Physical functioning	70.96(12.71)	70.50(12.98)	66.45(16.45)	66.86(13.28)	P=.166
Physical role	59.45(17.69)	56.31(21.70)	54.16(24.07)	49.78(16.54)	P=.090
Bodily pain	60.26(19.88)	59.76(17.29)	55.41(20.28)	50.52(15.85)	P=.094
General health	61.65(21.88)	56.50(21.96)	51.79(20.02)	48.30(16.73)	P=.007
Total physical health	62.83(15.91)	59.95(15.72)	55.70(18.35)	52.78(14.23)	P=.010
Vitality	57.47(18.26)	54.49(17.64)	49.16(18.74)	46.73(13.94)	P=.013
Social functioning	64.04(23.08)	59.39(23.17)	53.75(21.42)	49.34(17.94)	P=.007
Emotional role	59.60(27.69)	55.22(30.01)	54.37(25.24)	41.39(31.03)	P=.031
Mental wellbeing	65.61(19.38)	63.04(17.94)	59.75(16.78)	55.39(14.78)	P=.052
Total mental health	61.83(20.31)	57.84(20.60)	54.01(18.93)	48.30(16.99)	P=.008

Table 4 presents the result of linear regression analysis of the relationship between different types of domestic violence and HRQoL. Women who had experienced only one type of domestic violence did not have significantly lower scores in the SF-36 subscales (P>.05). Women who had experienced two types of violence had significantly lower scores in the one domain (total physical health) and the three subscales of SF-36 (‘general health’, ‘vitality’ and ‘social functioning’), and women who had experienced three types of violence had significantly lower scores in the two domains (‘total physical health’ and ‘total mental health’) and the seven subscales of SF-36 (‘physical role’, ‘bodily pain’, ‘general health’, ‘vitality’, ‘social functioning’, ‘emotional role’ and ‘mental wellbeing’) (P<.05). (Table 4)

**Table 4:** Regression analysis of the relationship between different types of domestic violence and HRQoL

Types of violence	One type of violence experienced N=99			Two types of violence experienced N=24			Three types of violence experienced N=23		
HRQoL subscales									
	B	95%CI	P	B	95%CI	P	B	95%CI	P
Physical functioning	-.34	-3.53; 2.88	.832	-4.76	-10.26; .746	.090	-5.09	-10.80; .622	.081

Physical role	-3.07	-7.86; 1.71	.208	-5.45	-13.60; 2.69	.189	-9.66	-18.12; -1.21	.025

Bodily pain	-.53	-5.20; 4.12	.820	-4.50	-12.44; 3.43	.265	-9.74	-17.97; -1.50	.021

General health	-5.05	-10.34; .23	.061	-10.05	-19.06; -1.04	.029	-13.34	-22.69; -4.00	.005

Total Physical health	-2.81	-6.74; 1.11	.159	-7.19	-13.88; -.51	.035	-10.05	-16.98; -3.12	.005

Vitality	-2.93	-7.33; 1.46	.191	-8.27	-15.76; -.78	.031	-10.73	-18.50; -2.96	.007

Social functioning	-4.56	-10.15; 1.03	.110	-10.44	-19.96; -.932	.032	-14.70	-24.57; -4.82	.004

Emotional role	-4.50	-11.53; 2.51	.208	-4.73	-16.68; 7.22	.437	-18.21	-30.62; -5.81	.004

Mental wellbeing	-2.50	-7.06; 2.05	.281	-6.01	-13.77; 1.74	.128	-10.22	-18.27; -2.17	.013

Total mental health	-3.94	-8.90; 1.00	.118	-7.83	-16.26; .59	.069	-13.53	-22.28; -4.78	.003

B: Unstandardized coefficient.

CI: Confidence Interval.

## 4. Discussion

The main objective of this study was to evaluate the association between domestic violence during pregnancy and women’s HRQoL. The present study revealed domestic violence during pregnancy was negatively related to the HRQoL of women after delivery, and both physical and mental health dimensions of HRQoL were significantly impaired in the women who had experienced domestic violence. Similarly, previous studies have reported impaired HRQoL among the abused women (Alsaker, Moen, Nortvedt, & Baste, 2006; Bonomi et al., 2006; Lau et al., 2008; Leung et al., 2005).

Our results showed that six of eight SF-36 subscales (except Physical functioning, and bodily pain) were significantly lower in the abused women compared with the non-abused women and the more the types of domestic violence experienced, the greater the impaired HRQoL. Consistent with our results, in a study by Lau et al. (2008) which was performed on 1200 postnatal women to examine the effect of intimate partner violence on HRQoL, women who had experienced different types of intimate partner violence reported significantly lower scores in the majority of the subscales of the SF-36, and there was a cumulative impact of intimate partner violence on the HRQoL of the women. Abused women appear to have limitations in physical activities and role functioning, cognitive impairment, and the negative perceptions of health that are likely to be linked to assault-related stress, impairing the functioning of the immune, endocrine, and autonomic systems, and can result in physical and mental health problems (Lau et al., 2008). Moreover, poor HRQoL can also be related to social factors and unhealthy lifestyles (Sørensen et al., 2012) such as alcohol and substance abuse which has been reported as a consequence of domestic violence (Audi et al., 2012).

In our study, physical functioning subscale had the highest score among the participants but was still lower in the abused women. This was also found in a study on Norwegian women who had experienced intimate partner violence. However, Alsaker et al. (2008) suggest that domestic violence has a considerable effect on women’s physical functioning (Alsaker et al., 2008). Thus, further research is needed to fully understand the impact of domestic violence on the physical dimension of HRQoL.

In this study, more than half of the abused women had experienced one type of domestic violence during pregnancy but women who had experience three types of violence were more likely to suffer impaired HRQoL. Fear and stress, resulting from exposure to the different types of domestic violence may induce the abused women to experience feelings of fatigue and weakness, reduced physical functioning and higher levels of depression. These women are also more likely to isolate themselves to avoid contact with relatives, friends or social groups (Lau et al., 2008). They may even be stigmatized by their family members for violating socio-cultural norms because of disclosure of domestic violence. Therefore, many abused Iranian women prefer to isolate themselves and not to ask for help from family and society due to fear for their own safety and future (Abadi et al., 2012).

The study results suggest that almost half of the participants had experienced domestic violence during pregnancy, mostly emotional, followed by sexual, then physical violence, which is a higher rate in comparison to the prevalence rate of 3 and 30 percent reported in other countries (Van Parys et al., 2014), indicating different cultural definitions of domestic violence in any society, the method of screening, and religious beliefs (Al-Atrushi, Al-Tawil, Shabila, & Al-Hadithi, 2013). Although, the rates of domestic violence in Iran are likely to be underreported due to expectations that women keep domestic violence a family secret (Abadi et al., 2012), our results are compatible with previous studies in Iran that reported domestic violence against pregnant women is common and emotional abuse is the most prevalent type of domestic violence among Iranian women (Pournaghash-Tehrani, 2011; Khodakarami, Naji, Dashti, & Yazdjerdi, 2009). Transforming the types of domestic violence, from the physical type to the emotional type is thought to be the result of socio-cultural changes taking place in Iran (Pournaghash-Tehrani, 2011). According to Tiwari et al. (2008), emotional abuse against pregnant women has a negative effect on their mental health after delivery (Tiwari et al., 2008). Women with emotional abuse may appear to function well, but the impact of the abuse on their overall health in general may be serious and long lasting (Lau et al., 2008). In fact, fear control and power play important roles in women’s negative consequences from domestic violence and are commonly associated with feelings of loneliness, isolation, helplessness and depression, impairing the women’s HRQoL (Wittenberg et al., 2007).

While the main finding of our study is the relationship between domestic violence during pregnancy and poor HRQoL, the causality of this association is unclear due to the cross-sectional design of the study, and given that impaired HRQoL may be antedated domestic violence. In fact, poor HRQoL is associated with poor functional status, fatigue, loss of energy, depression, restriction in activities and performance difficulties (Lau et al., 2008). Therefore, further studies are needed to compare the HRQoL of abused women before, during and after pregnancy through a longitudinal design. The findings of this study are also limited by the relatively small size of the sample, comprised entirely of women from urban health care centers and by relying on women’s self-reports which may have resulted in recall bias.

## 5. Conclusion

This study provides further evidence that domestic violence during pregnancy is negatively related to women’s HRQoL, and it should be considered as a major issue of public health priorities. In light of the findings of this study, the importance of screening pregnant and postnatal women for domestic violence, and the need for developing appropriate interventions to address domestic violence and improve HRQoL of abused women are emphasized. In addition, there is a need to educate health care providers regarding effective approaches to care for abused women.
